# Comparison of Pelvic Peritonectomy vs. Rectosigmoid Resection During Hudson Procedure for Advanced Ovarian Cancer: 6-Year Experience of an ESGO-Certified Center

**DOI:** 10.3390/cancers18030519

**Published:** 2026-02-05

**Authors:** Dimitrios Zouzoulas, Panagiotis Tzitzis, Iliana Sofianou, Katerina Tzika, Kimon Chatzistamatiou, Vasilis Theodoulidis, Eleni Timotheadou, Grigoris Grimbizis, Dimitrios Tsolakidis

**Affiliations:** 1 1st Department of Obstetrics & Gynecology, Aristotle University of Thessaloniki, “Papageorgiou” Hospital, 56429 Thessaloniki, Greece; 2 Department of Oncology, Aristotle University of Thessaloniki, “Papageorgiou” Hospital, 56429 Thessaloniki, Greece

**Keywords:** advanced ovarian cancer, Hudson procedure, pelvic peritonectomy, rectosigmoid resection

## Abstract

(1) Background: Hudson first described the procedure that includes en-block removal of an ovarian tumor fixed in the pelvis with the whole pelvic peritoneum and invaded surrounding structures. However, sometimes pelvic peritonectomy (PP) with or without shaving of the bowel serosa is not enough to achieve complete cytoreduction, and en-block rectosigmoid resection (RR) is necessary. This study aims to investigate the impact of bowel surgery on survival rates and morbidity of patients with advanced ovarian cancer. (2) Methods: We retrospectively analyzed patients with advanced ovarian cancer with cul-de-sac involvement that underwent debulking surgery at the 1st Department of Obstetrics—Gynecology of “Papageorgiou” General Hospital, from 2017–2022. The primary outcomes were the survival rates and morbidity between PP and RR. (3) Results: A total of 93 patients met the inclusion criteria. Patients were categorized into two groups: Group A (34 patients) with RR and Group B (59 patients) with PP. There was no statistically significant difference in the majority of patients’ characteristics and oncological outcomes. On the other hand, patients with RR had a significantly higher surgical complexity score (SCS), peritoneal cancer index (PCI), ICU admission, rate of postoperative complications, longer surgery duration and hospital stay. When comparing the duration of surgery, the RR group has significantly higher operation time during primary compared to interval debulking surgery. Concerning survival rates, there was no significant difference in progression-free (PFS) (*p* = 0.22) and overall survival (OS) (*p* = 0.85) between the two groups, while residual disease and postoperative complications were identified as independent prognostic factors for PFS and OS; (4) Conclusions: The modified Hudson procedure with RR is a safe and reproductible technique, but when complete gross resection can be achieved with PP, this technique is preferred in order to avoid increased patient’s morbidity.

## Simple Summary

This retrospective cohort compared pelvic peritonectomy (PP) versus rectosigmoid resection (RR) during the Hudson procedure in patients with advanced ovarian cancer and cul-de-sac involvement. Ninety-three women were treated in an ESGO-certified center between 2017 and 2022 were included and divided into RR (*n* = 34) and PP (*n* = 59) groups with comparable baseline characteristics and high complete gross resection rates (>70%). RR was more often performed in primary debulking and was associated with higher peritoneal cancer index (PCI) and surgical complexity score, longer operating time, increased ICU admission, longer hospitalization and higher overall postoperative complication rates, without an increase in major (grade III) events. Progression-free and overall survival did not differ significantly between the two groups. Multivariable Cox regression identified residual disease and total postoperative complication rate as independent prognostic factors for both survival endpoints.

## 1. Introduction

Ovarian cancer is the third most common gynecological malignancy and the leading cause of death among all women with genital tract cancers [1,2]. The majority of the patients are diagnosed at an advanced stage with high tumor load in the peritoneal cavity, leading to poorer survival rates [3,4]. Many prognostic factors have been proposed for the risk of recurrence or death. Residual disease after cytoreduction remains the most important prognostic factor [5,6], irrespective of surgery timing in relation to chemotherapy. Specifically, complete gross resection is the most important factor either in the primary [7,8], or in the interval [9,10,11], and even in the delayed setting [12,13]. Furthermore, recent data suggests that postoperative complications can independently affect survival rates irrespective of residual disease [14]. So, the goal of surgery is to achieve complete cytoreduction with the minimum morbidity for the patient in order to offer the best survival probability.

High tumor load in pelvis, even in cases of a “frozen pelvis”, during debulking surgery is no longer considered among gynecologist oncologists a contradiction to pursue a complete gross resection. Two techniques exist for pelvic cytoreduction. Rectosigmoid resection–anastomosis is a more extensive procedure associated with longer operative time, higher blood loss, stoma formation, and longer hospital stay compared with pelvic peritonectomy [15]. However, when complete cytoreduction is achieved, pelvic peritonectomy generally provides comparable oncologic outcomes to rectosigmoid resection, particularly in patients with superficial serosal involvement. The procedure, where the ovarian tumor that is fixed in the pelvis intact with the whole peritoneum and the surrounding structures still attached are removed en bloc, was first described by Christopher Hudson in 1968 [16] and further analyzed for its morbidity in 1973 [17]. This technique described the shaving of the rectosigmoid serosa, rather than its resection.

In the latter years, in order to unify terminology and to intergrade the need of rectosigmoid resection (RR) in some cases, a three-tier classification was proposed [18]. The procedure described by Hudson was classified as type I radical oophorectomy, where a complete pelvic peritonectomy (PP) with or without bowel serosa shaving is being performed. Type II radical oophorectomy, also known as modified Hudson procedure, consists of the previous procedure with the addition of an en bloc rectosigmoid resection. Lastly, type III radical oophorectomy is an extension of type II, as it includes the en bloc removal of a portion of the urinary bladder and/or the pelvic ureter. Furthermore, in order to standardize the Hudson procedure with PP, a 10-step approach has been developed [19], while critical anatomical tips and tricks have been described in detail [20]. Recently, a new anatomo-surgical classification for the modified Hudson procedure with RR has been proposed, assessing not only complete resection, but also the morbidity of the patients [21].

In the current era, the perception that the “frozen pelvis” in advanced ovarian cancer is considered manageable with bowel resection, and the fear of underestimation of the residual disease on the rectosigmoid serosa has led to a high rate (83.6%) of RR during cytoreduction [22]. However, data showed that intraoperative assessment of the depth of rectosigmoid wall involvement could be misleading, because histological examination reviled that up to 55% of patients undergoing RR during cytoreduction did not present full-thickness bowel wall infiltration [23]. These differences in surgical aggressiveness raise a clinical dilemma and justify a focused comparison between rectosigmoid resection and pelvic peritonectomy in advanced ovarian cancer. The aim of this study was to compare the impact of rectosigmoid resection and pelvic peritonectomy with or without bowel serosa shaving on the progression-free survival (PFS), overall survival (OS) and the morbidity of patients with advanced ovarian cancer.

## 2. Materials and Methods

### 2.1. Study Characteristics

For this retrospective analysis we reviewed the medical records of patients with ovarian cancer through the online registry system of the Gynecological–Oncology Unit of the 1st Department of Obstetrics & Gynecology, AUTh, “Papageorgiou” General Hospital, from 1 January 2017 until 31 December 2022. A total of 321 patients were diagnosed with ovarian cancer during this period of time. From these patients we identified those with a new diagnosis, those with an advanced stage disease [International Federation of Gynecology and Obstetrics (FIGO) stage III and IV] and finally those with involvement of the cul-de-sac. A written approval was received from the Institutional Review Board of the hospital.

### 2.2. Patients

#### 2.2.1. Inclusion Criteria

- Histologically confirmed ovarian cancer.
- Surgical treatment at our Gynecological–Oncology Unit.

#### 2.2.2. Exclusion Criteria

- Recurrent ovarian cancer.
- No macroscopic presence of disease in the cul-de-sac.
- Missing important survival data.

As a result of the above-mentioned criteria, 60 out of the 321 women with ovarian cancer were excluded due to early-stage disease. Moreover, 18 women were excluded, because of recurrent disease and 147 patients due to no evident disease in the cul-de-sac. Another 3 patients were removed from the cohort due to missing important survival data. Hence, finally 93 women with advanced ovarian cancer and involvement of the cul-de-sac were identified as eligible for further analysis.

All patients were preoperatively assessed with pelvic magnetic resonance imaging (MRI) to evaluate the presence and extent of bowel wall infiltration. Findings like loss of fat plane between pelvic mass and rectosigmoid wall, serosal thickening of the involved bowel segment, marked asymmetric wall thickening with submucosal edema and disruption or replacement of the muscularis propria by tumor were considered as an indication for RR [24,25]. Colonoscopy was not routinely performed—only if clinically suggested. The majority of the patients also underwent diagnostic laparoscopy, during which the rectosigmoid and the pelvic peritoneum were visualized in detail. Finally, during laparotomy itself the final decision about the necessity of PP or RR, in order to achieve complete gross resection, was made based on palpation and evaluation of the degree of infiltration and stenosis of the tumor through the bowel layers. Furthermore, patients with superficial implants larger than 2 cm or diffuse milliary disease in the rectosigmoid underwent RR. For both techniques, advanced bipolar energy devices were used (Ligasure Maryland jaw open sealer/divider), in order to minimize intra-operative blood loss [26]. For RR gastrointestinal stapling devices were used and end-to-end anastomosis were performed with circular stapler devices, at least about 6–10 cm from the anal verge. All rectosigmoid anastomoses were tested under water seal for any leakage with the “bubble test”.

All patients underwent debulking surgery combined with platinum-based chemotherapy, in the adjuvant or neoadjuvant setting, and the Enhanced Recovery After Surgery (ERAS) recommendations were followed [27,28], which were later also described by ESGO [29]. Specifically, avoidance of mechanical bowel preparation, and only rectal enemas the day before surgery were allowed, in combination with oral antibiotics. Preoperative fasting included intake of solids until midnight the day before surgery and a carbohydrate pre-loading drink at least two hours prior to surgery. All nasogastric tubes were removed before the reversal of the anesthesia, and placement of drains were left at the surgeons’ discretion, individualized for each case. Postoperatively, chewing gum was offered and early oral feeding were encouraged within the first 24 h after returning in the ward.

### 2.3. Data Collection

For this cohort of patients, all relevant data were collected during a period of 30 days from the online registry system of the Gynecological–Oncology Unit where all patient’s medical records are stored. In order to avoid differences among this long time period of data collection, a predefined data collection sheet (excel file) was used during the retrospective mining of the patient’s characteristics, oncological outcomes and survival data. The data sheet included the following information:

- Patient’s hospital identification number
- Patient’s age
- American Society of Anesthesiologists Physical Status Classification System (ASA) score
- Body Mass Index (BMI)
- Charlson Comorbidity Index (CCI)
- CA-125 level
- FIGO stage
- Intraoperative blood loss
- Surgery duration
- Surgical complexity score (SCS)
- Peritoneal cancer index (PCI)
- Histological types
- Intensive care unit (ICU) admission
- Clavien–Dindo classification for post-operative complications
- Hospital stay
- Type of pelvic surgery: RR or PP
- Stoma formation
- Residual disease (RD) after cytoreduction
- Type of cytoreduction: Primary or Interval
- Time related data:
- Date of surgery
- Date of recurrence or disease progression
- Date of last follow-up or death

Patients were divided into two groups based on the type of pelvic surgery during cytoreduction. Group A consisted of patients that underwent the modified Hudson procedure with rectosigmoid resection (RR), while patients in Group B underwent Hudson procedure with pelvic peritonectomy (PP) with or without bowel serosa shaving. Bowel shaving was defined as the tangential removal of superficial implants from the rectosigmoid without entering the lumen, while preserving the bowel mucosa wall integrity. The leading surgeon performing both surgical techniques was a senior professor of gynecological oncology, accredited by ESGO for advanced ovarian cancer surgery. The technique and the surgical steps that were followed in both procedures are described in detail in prior publications [19,20].

### 2.4. Statistical Analysis

Statistical analyses were performed with the R software, version 2025.09.0+387. For continuous variables, central tendency (mean, median), dispersion [interquartile range (IQR) and standard deviation (SD)] were calculated. On the other hand, for categorical variables, descriptive statistics were expressed as absolute frequencies and percentages. Progression-free (PFS) and overall survival (OS) analyses were calculated using the Kaplan–Meier curves, and the Cox regression was used for univariable and multivariable analysis. Progression-free survival was defined as the time period between date of surgery and date of first recurrence or disease progression, while overall survival as the time period from surgery to the date of death or last follow-up. A *p*-value of <0.05 was considered as statistically significant.

## 3. Results

Initially, 321 women with ovarian cancer that underwent surgery in the Gynecological–Oncology Unit, 1st Department of Obstetrics & Gynecology, Aristotle University of Thessaloniki, “Papageorgiou” General Hospital, were included in this retrospective cohort. After screening the patients based on the inclusion and exclusion criteria, 93 patients with advanced-stage disease and involvement of the cul-de-sac were eligible for further analysis.

Patients’ characteristics are outlined in Table 1. The population of the study was over-weighted, with mild to moderate comorbidities and a median age of 61 years old. The majority of the patients had high-grade serous FIGO stage III ovarian cancer and underwent interval debulking surgery. Concerning the type of pelvic surgery, in over two-thirds of the population PP with or without bowel serosa shaving was offered. Moreover, the median surgery duration and intraoperative blood loss were 300 min and 400 cc, respectively. The majority of the patients (85%) underwent intermediate- and high-complexity cytoreduction, with a median SCS and PCI of 6 and 13, respectively. Postoperative morbidity showed that one-third of the patients needed ICU admission, with only 8.6% of major complications and a median hospital stay of 8 days. Complete gross resection rates were high, up to 72%, and in cases of recurrence the site of relapse was rarely located in the pelvis (13%). Furthermore, concerning patients in Group A with rectosigmoid resection and end-to-end anastomosis, a prophylactic stoma was performed only in six cases (17.6%), and no significant association with major postoperative complications (≥grade III) was found (stoma: 16.7% vs. no stoma: 10.7%, *p*-value = 0.5585).

Based on the type of pelvic surgery, the population of this cohort was divided into two groups: Group A with 34 patients (36.6%) who underwent in the pelvis the modified Hudson procedure with rectosigmoid resection (RR), and Group B with 59 patients (63.4%) who underwent in the pelvis the Hudson procedure with pelvic peritonectomy (PP) with or without bowel serosa shaving. The two groups were similar in terms of baseline characteristics. No statistically significant differences were found in age, ASA score, BMI, comorbidities, CA-125 pre-operative values, FIGO stage and histological subtypes, suggesting homogeneity between the two groups. Concerning surgical outcomes, they were comparable in intraoperative blood loss, residual disease and the site of recurrence. Specifically, in both groups complete gross resection rates were as high as 70% and local disease control in the pelvis was satisfactory, with a low pelvic recurrence rate of 13% for both procedures. On the other hand, the rate of RR was statistically significantly higher during primary debulking surgery, and a statistically significant difference was observed in surgery duration (RR: 390 min vs. PP: 300 min), peritoneal tumor dissemination (RR PCI: 17 vs. PP PCI: 11) and surgical complexity score (RR SCS: 8 vs. PP SCS: 5). Furthermore, a statistically significant increase in the morbidity was discovered in the RR group, as well as a higher rate of ICU admission (RR: 58.8% vs. PP: 22%), a longer hospital stay (RR: 9 vs. PP: 8) and an increased total postoperative complication rate (RR: 32 vs. PP: 23.4), but with no difference in major events (≥grade III complications). The aforementioned data are presented in detail in Table 2.

**Figure 1:**
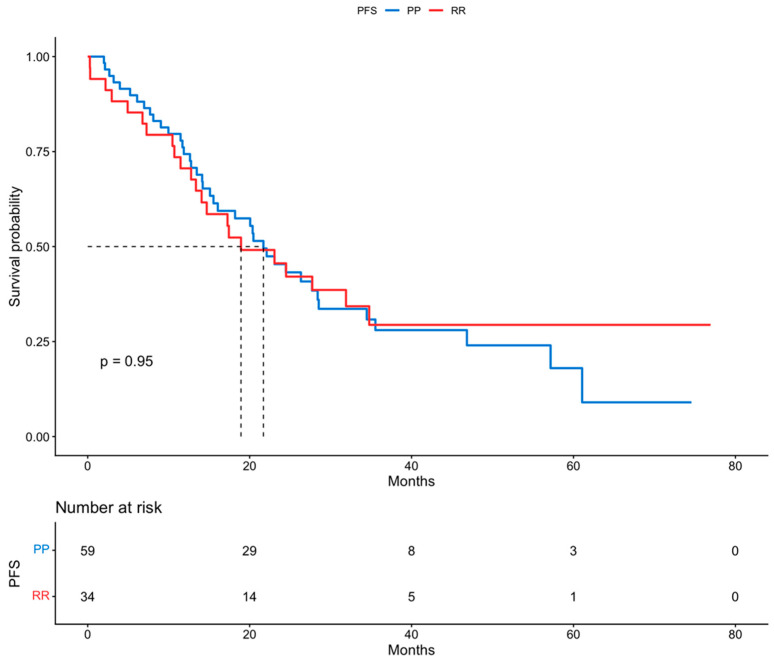
Progression-free survival (Kaplan–Meier curve).

**Figure 2:**
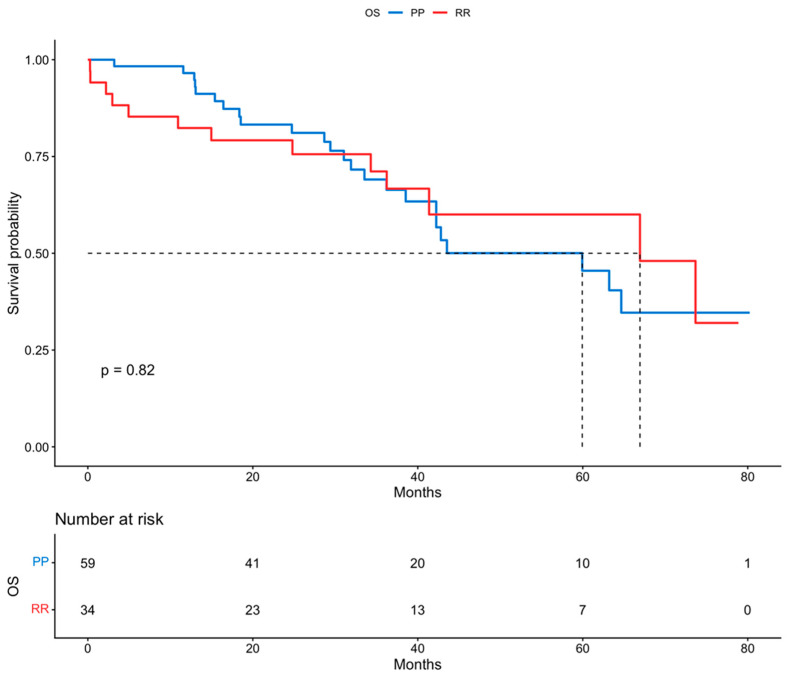
Overall survival (Kaplan–Meier curve).

All patients included in this cohort had frequent routine review visits, with a long mean follow-up period of 34.7 months. Survival outcomes were estimated using Kaplan–Meier curves. In the analysis between the two groups (RR vs. PP), the median progression-free survival (PFS) in Group A with rectosigmoid resection and Group B with pelvic peritonectomy was 19 and 22 months, respectively. Moreover, the median overall survival (OS) for Group A and Group B was 67 months and 60 months, respectively. By using log-rank tests, no statistically significant difference was found in PFS (*p* = 0.95) and also in OS (*p* = 0.82) between the two groups. These results are presented in Figure 1 and Figure 2.

The type of pelvic surgery (RR vs. PP) during Hudson procedure at cytoreduction for advanced ovarian cancer did not influence either PFS or OS. So, we further investigated the possible prognostic factors affecting the survival rates of patients with advanced ovarian cancer. In order to identify these factors, a univariable and multivariable analysis was conducted, including all the parameters that were collected for patients of this cohort in order to avoid the effect of the confounders.

Initially, a Cox regression for the risk of recurrence or disease progression was performed. In the univariable analysis a statistical significance was observed in total postoperative complication rate and residual disease. Continuing with the multivariable analysis, factors with a *p*-value < 0.2 were included. Total postoperative complication rate, measured with the Clavien–Dindo classification, and any residual disease after cytoreduction were identified as independent prognostic factors for PFS in advanced ovarian cancer patients. Interestingly, for each 1-point increase in the Clavien–Dindo classification, the hazard of recurrence or disease progression was increased by 3% (HR: 1.03, 95%CI: 1.01, 1.05, *p*-value < 0.05). In addition, when optimal cytoreduction was achieved, the risk of recurrence or disease progression was approximately 2.5-fold higher than complete gross resection (HR: 2.71, 95%CI: 1.54, 4.75, *p*-value < 0.05), while in suboptimal cytoreduction the risk was approximately 15-fold higher (HR: 14.69, 95%CI: 4.09, 52.77, *p*-value < 0.05). The aforementioned data are detailed presented in Table 3.

Furthermore, a Cox regression for the risk of death was performed. In the univariable analysis, statistical significance was observed in total postoperative complication rate and residual disease. Continuing the multivariable analysis including factors with a *p*-value < 0.2, both total postoperative complication rate, measured with the Clavien–Dindo classification, and any residual disease after cytoreduction were identified as independent prognostic factors for OS in advanced ovarian cancer patients. In detail, for each 1-point increase in the Clavien–Dindo classification, the hazard of recurrence or disease progression was increased by 4% (HR: 1.04, 95%CI: 1.02, 1.07, *p*-value < 0.05). In addition, when optimal cytoreduction was achieved, the risk of recurrence or disease progression was approximately three-fold higher compared to complete gross resection (HR: 3.30, 95%CI: 1.56, 6.55, *p*-value < 0.05), while in suboptimal cytoreduction the risk was approximately 30-fold higher (HR: 28.05, 95%CI: 6.72, 117.07, *p*-value < 0.05). These results are presented in Table 4.

## 4. Discussion

The primary endpoint of our study was to investigate the impact of rectosigmoid resection and pelvic peritonectomy with or without bowel serosa shaving during Hudson procedure on the survival rates and morbidity of ovarian cancer patients. Initially, patients with advanced ovarian cancer and involvement of the cul-de-sac were identified. All of them followed the ERAS recommendations, specifically, no mechanic bowel preparation and only rectal enemas were allowed, in combination with oral antibiotics, while carbohydrate pre-loading at least two hours prior to surgery was encouraged. However, it is important to state that recently published guideline from the American Society of Colon and Rectal Surgeons (ASCRS) contradict ERAS recommendations and strongly recommend that oral antibiotics in combination with mechanical bowel preparation have been shown to decrease the incidence of surgical site infection after elective colorectal resection [30]. Based on the type of pelvic surgery, sparing or not of the rectosigmoid, the population of the cohort was divided into two groups: Group A (rectosigmoid resection) with 34 (36.6%) and Group B (pelvic peritonectomy) with 59 (63.4%) patients.

The two groups were homogenous in their baseline characteristics, allowing robust between-group comparisons. However, tumor load differed by study design between the two groups, where low-burden resectable disease was more frequent in the PP group. Most patients in our cohort had high-grade serous FIGO stage III ovarian cancer and underwent interval debulking surgery, reflecting the contemporary shift toward neoadjuvant chemotherapy (NACT) in advanced disease and underlining the need to interpret these pelvic procedures within this treatment context. Concerning surgical outcomes, the majority of patients with RR had a higher PCI and underwent primary debulking surgery with high SCS and surgery duration. However, patients with RR showed a statistically significant ICU admission rates, hospital stay and total postoperative complications (without statistically significant difference in ≥grade III events). This indicates that when PP was performed and the rectosigmoid was spared, patients showed less short-term morbidity. The rate of prophylactic stoma during end-to-end anastomosis was low (17.6%) and was not associated with less postoperative morbidity. Routine stoma formation should be avoided and individualized case per case. Residual disease showed no difference between the two groups, highlighting the importance of correct patient triage to undergo either RR or PP during Hudson procedure.

Furthermore, univariable and multivariable analyses showed that residual disease and postoperative complications are independent prognostic factors for PFS and OS. These results further emphasize the importance of selecting the correct pelvic technique during the Hudson procedure for cul-de-sac involvement, at the correct setting (primary vs. interval). Even though NACT was not a statistically significant covariate for recurrence or death in univariable and multivariable analysis, our data show that the majority of the patients (76.3%) underwent PP in the interval setting. This finding suggests a possible effect of NACT in the pelvic procedure to achieve complete gross resection. Rectosigmoid resection (Group A) should not always be the gold-standard, especially when complete gross resection could be achieved with pelvic peritonectomy with or without bowel serosa shaving (Group B). The more cost-effective approach of pelvic peritonectomy, even with a discoid resection for a limited, typically antimesenteric, full-thickness infiltration of the bowel wall, should always be first pursued in order to avoid a formal full rectosigmoid resection–anastomosis, which is associated with increased surgical complexity, longer operative time, higher complication rates and prolonged hospital stay (with related costs), without clearly demonstrating additional oncological benefit.

To our knowledge there are only a few studies in the literature that compare the Hudson and the modified Hudson procedure. The first retrospective study from Aletti et al. [31] included 128 patients that underwent either RR or PP. Similarly to our results, the authors found no difference between the two groups concerning major complications, but in contrast, the type of pelvic surgery (RR vs. PP) had a significant impact on OS when complete gross resection was achieved (patient with RR had an improved OS). Residual disease and type of pelvic surgery were identified as independent prognostic factors, after multivariable analysis. The authors hypothesize that this could be a result of pelvic peritoneum or bowel serosa RD underestimation. Additionally, the lack of data for recurrence and the small sample size (*n* = 25) of the subgroup survival analysis raise questions about the validity of the results.

Furthermore, two single-center retrospective studies from 2011 and 2018 also investigated the role of rectosigmoid resection and pelvic peritonectomy. Gallotta et al. [32] in 2011 included 187 patients with advanced ovarian cancer and found no significant difference in the survival rates and major complications between the two groups, which is in accordance with our results. Multivariable analysis identified only RD as an independent prognostic factor. Concerning prophylactic stomas, the authors state that the rate of 46.5% was significantly less as the studied period progressed, concluding that routine stoma formation should be avoided, which is agreement with our results. In contrast to our findings, the PP group had a higher rate of pelvic recurrences compared to the RR group. The authors stated that this could be attributed to underestimation of RD in pelvic peritoneum or rectosigmoid serosa. This difference could also be explained by the fact that in our study, solitary superficial rectosigmoid implants were excised “bowel serosa shaving”, along with pelvic peritonectomy. Erkilinç et al. [33] in 2018 included 178 patients with cul-de-sac involvement, but excluded patients with deep muscular rectosigmoid infiltration or patients that needed “bowel serosa shaving” according to criteria applied to our study. No statistically significant difference was observed between the two groups concerning PFS, OS, postoperative major complications and pelvic recurrences, all in alignment with the results of our study. In contrast, only >1000 cc of ascites and primary cytoreduction were independent prognostic factor for OS and none for PFS.

The most recent retrospective study from Japan [34] included 84 patients with advanced ovarian cancer that underwent either rectosigmoid resection or pelvic peritonectomy. Similarly to our findings, no statistically significant difference was found in PFS, OS, major postoperative complications and pelvic relapses between the two groups. Moreover, the authors state that a multivariable analysis for the survival rates was conducted, but no clear data about the univariate analysis and the included confounders were reported. It is important to state that in contrast to our study, all four previous studies in the literature did not use the Clavien–Dindo classification to calculate the total postoperative complication rate. Moreover, the most recent study in 2025 that investigated this topic was a systematic review and meta-analysis [15]. The authors concluded that there was no statistically significant difference in terms of survival rates (DFS and OS) and pelvic recurrence rates. Pelvic peritonectomy was associated with less morbidity compared to rectosigmoid resection. These results are in total agreement with the findings of our study.

The prognostic factors for advanced ovarian cancer patients’ survival rates that were identified in our study are in complete agreement with previous publications. Residual disease is the most important independent prognostic factor for survival in the primary, as established recently with the TRUST trial [8], as well as in the interval, established with three landmark trials [9,10,11], and also in the delayed setting, established in recently published studies [12,13]. Furthermore, total postoperative complication rate, calculated with the Clavien–Dindo classification, was recently identified as an independent prognostic factor for survival in a large, real-world multicenter study [14].

The strengths of our study included the long follow-up period and the implementation of multivariate analyses with many possible confounders. This was the first study in the literature assessing the peritoneal tumor load and dissemination with PCI, the extent-complexity of surgery with SCS and the postoperative complications with the Clavien–Dindo classification, allowing better statistical analyses of the results and future reproducibility. Furthermore, our department is a university tertiary ESGO certified for advanced ovarian cancer surgery, ensuring a high-level of quality indicators and surgical proficiency. This is also confirmed by the high (>70%) complete gross resection rate of the population of our study. In contrast, the main limitations are the retrospective nature of the study and its relatively small sample size. A possible selection bias could be argued, because by design, cases with less tumor load were assigned to the PP group, and no subgroup analysis based on the administration of NACT was performed. Additionally, the lack of data about BRCA/HRD status could possibly be a constraint.

The principal contribution of this study to the literature was the implementation of the ERAS recommendations in patients undergoing modified Hudson procedure with RR and its promising results showing no difference in major (≥grade III) postoperative complications.

Future large, randomized control trials should stratify outcomes not only by the type of pelvic procedure, but also by the use and number of NACT cycles, to better disentangle the impact of chemotherapy-induced pelvic changes from the intrinsic morbidity of rectosigmoid resection versus pelvic peritonectomy. Moreover, the ongoing study “Comparison of Rectosigmoid Resection and Seromuscular Tumor Shaving Methods in Ovarian Cancer Surgery (BROSEOC), NCT04665635” could confirm the above-mentioned results. The role of pelvic peritonectomy should be also investigated in a prospective setting for early-stage ovarian cancer patients, especially because retrospective data [35] showed promising results with lower pelvic recurrence rates.

## 5. Conclusions

During Hudson procedure, the rectosigmoid resection is a safe and reproductible technique for pelvic surgery, while pelvic peritonectomy is an oncologically safe, less radical option in carefully selected, lower-burden cases. When complete gross resection can be achieved with pelvic peritonectomy with or without bowel serosa shaving, this technique should be preferred in order to avoid increased patient morbidity with no survival benefit. Properly chosen patients to undergo RR during primary cytoreduction have the same survival benefit as patients that undergo PP during interval cytoreduction. Residual disease and total postoperative complication rate are identified as independent prognostic factors for progression-free and overall survival for advanced-stage ovarian cancer patients with cul-de-sac involvement.

## Figures and Tables

**Table 1:** Baseline characteristics.

		Number of Patients (N)	Percentage (%)
Age (years)		mean: 60.7	SD: 11.6
BMI (kg/m^2^)		median: 27.2	IQR: 23.7–31.2
ASA score		median: 2	IQR: 1–2
CCI		median: 3	IQR: 2–4
	0–2	35	37.6
	3–4	37	39.8
	≥5	21	22.6
CA-125 (U/mL)		median: 79.8	IQR: 24.3–507.5
FIGO stage			
	III	72	77.4
	IV	21	22.6
Histological subtype			
	serous	81	87.1
	other	12	12.9
Type of cytoreduction			
	primary	32	34.4
	interval	61	65.6
Type of pelvic surgery			
	RR	34	36.6
	PP	59	63.4
Intraoperative blood-loss (mL)		median: 400	IQR: 300–500
Surgery duration (min)		median: 300	IQR: 240–400
SCS		median: 6	IQR: 4–8
	low: 1–3	14	15
	intermediate: 4–7	49	52.7
	high: ≥8	30	32.3
PCI		median: 13	IQR: 9–19
ICU admission			
	yes	33	35.5
	no	60	64.5
Clavien–Dindo classification		median: 25.7	IQR: 18.4–33.2
	grade I–II	85	91.4
	≥grade III	8	8.6
Stoma formation (Group A)			
	yes	6	17.6
	no	28	82.4
Hospital stay (days)		median: 8	IQR: 8–11
Residual disease (cm)			
	0	67	72
	<1	23	24.7
	≥1	3	3.3
Recurrence site			
	local	6	13
	distant	15	87

**Table 2:** Comparison based on type of pelvic surgery.

Characteristics		Group A (RR) 34 (36.6%)	Group B (PP) 59 (63.4%)	*p*-Value
Age (years) mean (SD)		63.4 (10.6)	59.2 (12)	0.092
BMI (kg/m^2^) median (IQR)		26.8 (24.5–30.7)	27.4 (23.6–31.2)	0.888
ASA score median (IQR)		2 (1–2)	2 (1–2)	0.851
CCI median (IQR)		3 (2–5)	3 (2–4)	0.140
CA-125 (U/mL) median (IQR)		189.2 (39.9–538.1)	41.6 (17.3–507.5)	0.076
FIGO stage				0.449
	III	28 (82.4%)	44 (74.6%)	
	IV	6 (17.6%)	15 (25.4%)	
Histological subtypes				0.05004
	serous	33 (97.1%)	48 (81.4%)	
	other	1 (2.9%)	11 (18.6%)	
Type of cytoreduction				**<0.05**
	primary	18 (52.9%)	14 (23.7%)	
	interval	16 (47.1%)	45 (76.3%)	
Intraoperative blood-loss (mL) median (IQR)		450 (300–625)	300 (300–500)	0.099
Surgery duration (min) median (IQR)		390 (323–426)	300 (240–360)	**<0.05**
SCS median (IQR)		8 (8–9)	5 (4–6)	**<0.05**
PCI median (IQR)		17 (11.25–21.5)	11 (8–17)	**0.032**
ICU admission				**<0.05**
	yes	20 (58.8%)	13 (22%)	
	no	14 (41.2%)	46 (78%)	
Clavien–Dindo classification median (IQR)		32 (31.7–38.2)	23.4 (12.2–32)	**<0.05**
Clavien–Dindo classification				0.1623
	grade I–II	30 (88.2%)	55 (93.2%)	
	≥grade III	4 (11.8%)	4 (6.8%)	
Hospital stay (days) median (IQR)		9 (8–13)	8 (7–10)	**0.014**
Residual disease (cm)				0.6429
	0	25 (73.5%)	42 (71.2%)	
	<1	9 (26.5%)	14 (23.7%)	
	≥1	0 (0%)	3 (5.1%)	
Recurrence site				1.00
	local	2 (12.5%)	4 (13.3%)	
	distant	14 (87.5%)	26 (86.7%)	

**Table 3:** Cox regression for recurrence or disease progression.

		Univariate			Multivariate		
		HR	95% CI	*p*-Value	HR	95% CI	*p*-Value
Age (years)		1.02	0.99, 1.04	0.0725			
ASA score		1.30	0.90, 1.87	0.17			
BMI (kg/m^2^)		1.01	0.98, 1.02	0.324			
CCI		1.13	0.99, 1.28	0.0731			
CA-125 (U/mL)		1.00	1.00, 1.01	0.0675	1.00	0.99, 1.01	0.293
FIGO stage							
	III	1	1	1			
	IV	1.37	0.75, 2.51	0.308			
Histological subtypes							
	serous	0.92	0.45, 1.87	0.81			
	other	1	1	1			
Type of cytoreduction							
	primary	0.74	0.43, 1.27	0.281			
	interval	1	1	1			
Type of pelvic surgery							
	RR	1	1	1			
	PP	1.02	0.60, 1.72	0.948			
Intraoperative blood-loss (mL)		1.00	0.99, 1.01	0.194			
Surgery duration (min)		0.99	0.99, 1.01	0.704			
SCS		0.99	0.90, 1.11	0.962			
PCI		1.03	0.99, 1.07	0.0996	0.94	0.88, 1.01	0.0816
ICU admission							
	yes	0.96	0.56, 1.62	0.864			
	no	1	1	1			
Clavien–Dindo classification		1.03	1.01, 1.05	**<0.05**	1.03	1.01, 1.05	**<0.05**
Hospital stay (days)		1.01	0.97, 1.07	0.578			
Residual disease (cm)							
	0	1	1	1	1	1	1
	<1	2.89	1.67, 5.02	**<0.05**	2.71	1.54, 4.75	**<0.05**
	≥1	11.24	3.25, 38.90	**<0.05**	14.69	4.09, 52.77	**<0.05**

**Table 4:** Cox regression for death.

		Univariate			Multivariate		
		HR	95% CI	*p*-Value	HR	95% CI	*p*-Value
Age (years)		1.02	0.99, 1.05	0.239			
ASA score		1.14	0.68, 1.92	0.618			
BMI (kg/m^2^)		1.05	0.97, 1.13	0.258			
CCI		1.13	0.95, 1.34	0.182	1.14	0.96, 1.34	0.130
CA-125 (U/mL)		1.00	0.99, 1.01	0.204			
FIGO stage							
	III	1	1	1			
	IV	1.51	0.67, 3.40	0.315			
Histological subtypes							
	serous	0.99	0.39, 2.55	0.985			
	other	1	1	1			
Type of cytoreduction							
	primary	0.73	0.37, 1.47	0.383			
	interval	1	1	1			
Type of pelvic surgery							
	RR	1	1	1			
	PP	1.08	0.55, 2.13	0.822			
Intraoperative blood-loss (mL)		1.00	0.99, 1.01	0.265			
Surgery duration (min)		1.00	0.99, 1.01	0.84			
SCS		0.93	0.81, 1.06	0.267			
PCI		1.03	0.98, 1.08	0.264			
ICU admission							
	yes	1.09	0.55, 2.19	0.801			
	no	1	1	1			
Clavien–Dindo classification		1.04	1.01, 1.06	**<0.05**	1.04	1.02, 1.07	**<0.05**
Hospital stay (days)		0.98	0.91, 1.07	0.692			
Residual disease (cm)							
	0	1	1	1	1	1	1
	<1	3.25	1.63, 6.49	**<0.05**	3.20	1.56, 6.55	**<0.05**
	≥1	23.94	5.95, 96.37	**<0.05**	28.05	6.72, 117.07	**<0.05**

## Data Availability

In accordance with the journal’s guidelines, the data presented in this study are available on request from the corresponding author for the reproducibility of this study if such is requested.
